# Improved In Vivo Efficacy of Anti-Hypertensive Biopeptides Encapsulated in Chitosan Nanoparticles Fabricated by Ionotropic Gelation on Spontaneously Hypertensive Rats

**DOI:** 10.3390/nano7120421

**Published:** 2017-12-02

**Authors:** Shehu Muhammad Auwal, Mohammad Zarei, Chin Ping Tan, Mahiran Basri, Nazamid Saari

**Affiliations:** 1 Department of Food Science, Faculty of Food Science and Technology, Universiti Putra Malaysia, Serdang 43400, Selangor, Malaysia; samuhammad.bch@buk.edu.ng (S.M.A.); mzarei.mail@gmail.com (M.Z.); 2 Department of Biochemistry, Faculty of Basic Medical Sciences, Bayero University, Kano 700231, Nigeria; 3 Department of Food Science and Technology, College of Agriculture and Natural Resources, Sanandaj Branch, Islamic Azad University, Sanandaj 66131, Iran; 4 Department of Food Technology, Faculty of Food Science and Technology, Universiti Putra Malaysia, Serdang 43400, Selangor, Malaysia; tancp@upm.edu.my; 5 Department of Chemistry, Faculty of Science, Universiti Putra Malaysia, Serdang 43400, Selangor, Malaysia; mahiran@upm.edu.my

**Keywords:** angiotensin I-converting enzyme, ACE-inhibitory biopeptides, blood pressure, oral administration, enzymatic degradation, bioavailability, efficacy, chitosan nanoparticles, physicochemical properties, spontaneously hypertensive rats

## Abstract

Recent biotechnological advances in the food industry have led to the enzymatic production of angiotensin I-converting enzyme (ACE)-inhibitory biopeptides with a strong blood pressure lowering effect from different food proteins. However, the safe oral administration of biopeptides is impeded by their enzymatic degradation due to gastrointestinal digestion. Consequently, nanoparticle (NP)-based delivery systems are used to overcome these gastrointestinal barriers to maintain the improved bioavailability and efficacy of the encapsulated biopeptides. In the present study, the ACE-inhibitory biopeptides were generated from stone fish (*Actinopyga lecanora*) protein using bromelain and stabilized by their encapsulation in chitosan (chit) nanoparticles (NPs). The nanoparticles were characterized for in vitro physicochemical properties and their antihypertensive effect was then evaluated on spontaneously hypertensive rats (SHRs). The results of a physicochemical characterization showed a small particle size of 162.70 nm, a polydispersity index (pdi) value of 0.28, a zeta potential of 48.78 mV, a high encapsulation efficiency of 75.36%, a high melting temperature of 146.78 °C and an in vitro sustained release of the biopeptides. The results of the in vivo efficacy indicated a dose-dependent blood pressure lowering effect of the biopeptide-loaded nanoparticles that was significantly higher (*p* < 0.05) compared with the un-encapsulated biopeptides. Moreover, the results of a morphological examination using transmission electron microscopy (TEM) demonstrated the nanoparticles as homogenous and spherical. Thus, the ACE-inhibitory biopeptides stabilized by chitosan nanoparticles can effectively reduce blood pressure for an extended period of time in hypertensive individuals.

## 1. Introduction

Hypertension is defined as a sustained increase in systolic blood pressure greater than 140 mmHg and/or diastolic blood pressure less than 90 mmHg. It is inferred as a common public health risk of global concern that is associated with cardiovascular-related diseases such as heart failure, kidney failure, stroke and disability [1,2].

Cardiovascular diseases (CVDs) have been identified as the major cause of mortality, with a global death rate of greater than 17.3 million cases in 2013, which accounted for 31% of all the reported deaths during the year [3,4]. The rate of death incidence due to poorly controlled CVDs is expected to exceed 23.6 million worldwide by 2030 [5]. However, it has been previously projected that a 34% decrease in premature death due to CVDs in the vulnerable group (30–70 years) could be achieved by the year 2025 through specifically targeting smoking, alcoholism, obesity, elevated blood pressure, glucose and salt intake as the major risks factors [6].

More importantly, the suppression of hypertension/elevated blood pressure alone among other risk factors could sufficiently reduce mortality due to CVDs by 30.4% and 38.0% in males and females, respectively [7].

Presently, ACE-inhibitors, renin inhibitors, aldosterone inhibitors, beta blockers and angiotensin receptor blockers are the five major classes of antihypertensive drugs with specific target site inhibitory action on the renin–angiotensin–aldosterone system (RAAS) that are used in the management of hypertension. However, the therapeutic potential of such drugs is limited by their cost and related adverse effects, including insulin resistance and diabetes, skin rashes and taste disturbance, etc. [8,9].

Alternatively, ACE-inhibitory biopeptides generated through the enzymatic hydrolysis of food proteins have been reported to exhibit blood-pressure-lowering capacities in hypertensive animals with little or no side effects [10,11,12,13]. In contrast to synthetic drugs, food-derived antihypertensive biopeptides can be cheaply produced from certain proteins obtained from locally available food sources. However, gastrointestinal digestion and poor intestinal permeation lead to the low bioavailability and duration of action of orally-administered biopeptides [14,15].

Several approaches including permeation enhancers, enzyme inhibitors and different polymeric carrier systems have been used to overcome these challenges [15,16]. In this regard, nanoencapsulation become the most desirable approach to protect the biopeptides against digestive enzymes, enhance their cellular uptake and control their release for effective oral-colon delivery and biological efficacy [15,16,17].

By virtue of their large surface-area-to-volume ratio, small-sized nanocapsules have been reported to circumvent first pass effects and to improve the solubility, gastrointestinal stability, target site delivery, bioavailability and biological role of the encapsulated bioactive agent [18]. In due course, nanoparticles prolong the half-life or retention time and reduce the dosing frequency of the incorporated antihypertensive peptides and proteins [19].

Chitosan has been safely used as a pH sensitive, biodegradable, biocompatible and mucoadhesive polymer in the fabrication of polymeric nanoparticles for oral-colon delivery and the controlled release of bioactive peptides and proteins [20,21,22].

Chitosan-based nanoparticles have been reported to enhance the oral delivery and bioavailability of anti-cancer agents [23,24,25,26] and antidiabetic agents [27], among others.

In the present study, chitosan nanoparticles were produced and evaluated as potential oral delivery vehicles for the ACE-inhibitory biopeptides derived from stone fish protein using bromelain. The biopeptides were nanoencapsulated using chitosan by the ionotropic gelation method and characterized in vitro for physicochemical properties. The antihypertensive efficacy of the chitosan nanoparticles containing biopeptides was then studied on spontaneously hypertensive rats.

## 2. Results and Discussion

### 2.1. Optimization of Process Variables for the Preparation of Chit-AntBiop-NPs

The Chitosan-AntBiop-NPs were fabricated by a simple technique [21] under optimum conditions of AntBiop:Chitosan mass ratio, stirring speed and stirring time. The effects due to these factors on particle size and encapsulation efficiency were studied to achieve stable and small-sized nanocapsules with high entrapment efficiency (EE).

**Figure 1:**
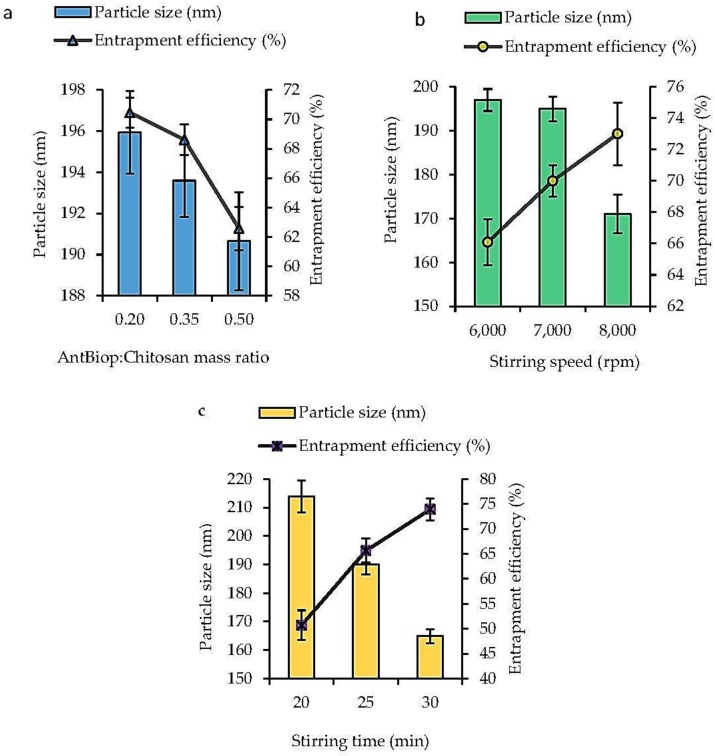
Effect of formulation parameters on the particle size (nm) and entrapment efficiency (EE, %) of Chit-AntBiop-NPs (**a**) AntBiop:Chitosan mass ratio; (**b**) stirring speed; (**c**) and stirring time.

As shown in Figure 1a, both the particle size and EE were negatively correlated with the AntBiop:Chitosan mass ratio within the range of 0.2 to 0.5.

Figure 1b demonstrated that the particle size decreased while the EE increased with an increase in stirring speed from 6000 to 8000 rpm.

In Figure 1c, the particle size was inversely related to the increase in stirring time, whereas the EE increased with an increased stirring time within the range of 20 to 30 min.

**Figure 2:**
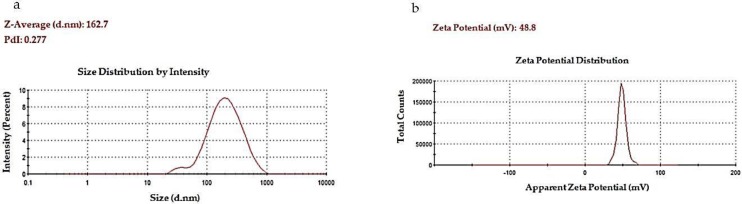
Physical properties of Chit-AntBiop-NPs; (**a**) particle size and pdi, (**b**) zeta potential.

The optimized condition to fabricate the Chitosan-AntBiop-NPs was established to include a AntBiop:Chitosan mass ratio of 0.35, stirring speed of 8000 rpm and a stirring time of 25 min. The Chitosan-AntBiop-NPs prepared under these conditions showed a high EE of 75.36%. The NPs also have a small particle size of 162.70 nm, a low pdi value of 0.28, and a high positive zeta potential (colloidal stability) of 48.78 mV (Figure 2).

The increased chitosan concentration caused a decreased shear effect, which lead to a highly viscous formulation characterized by capsules of large particle size and high EE. However, the tween 80 that was used as an emulsifier to sterically stabilize the nanocapsules induced reduced surface tension and improve the shear effect, resulting in smaller-sized nanocapsules while still maintaining a high EE.

As previously reported, the size of the chitosan nanoparticles increased with the increase in concentration of chitosan [28], and a high entrapment efficiency was achieved at low protein loading and higher chitosan concentration [29]. Similarly, the decrease in the particle size due to inclusion of tween 80 in the formulation was also previously observed [30,31].

The stirring speed and stirring time could affect particle size and the rate at which chitosan formed a viscous gel to ionically gelate the AntBiop. Thus, the increase in these factors and the amount of cross-linking agent, sodium tripolyphosphate, were found to maximize the incorporation and the entrapment efficiency of the AntBiop in the Chit-AntBiop-NPs and to lead to smaller sized nanocapsules. In a similar finding, the stirring speed and stirring time were optimized to produce chitosan nanoparticles with a small particle size and high EE [32]. However, a prolonged stirring rate beyond the optimum point may lead to leakage of the AntBiop and low EE [33].

In contrast, we were able to fabricate nanocapsules of much smaller particle size with high entrapment efficiency and less stirring time.

### 2.2. Characterization of Chitosan-AntBiop-NPs

#### 2.2.1. Transmission Electron Microscopy (TEM)

**Figure 3:**
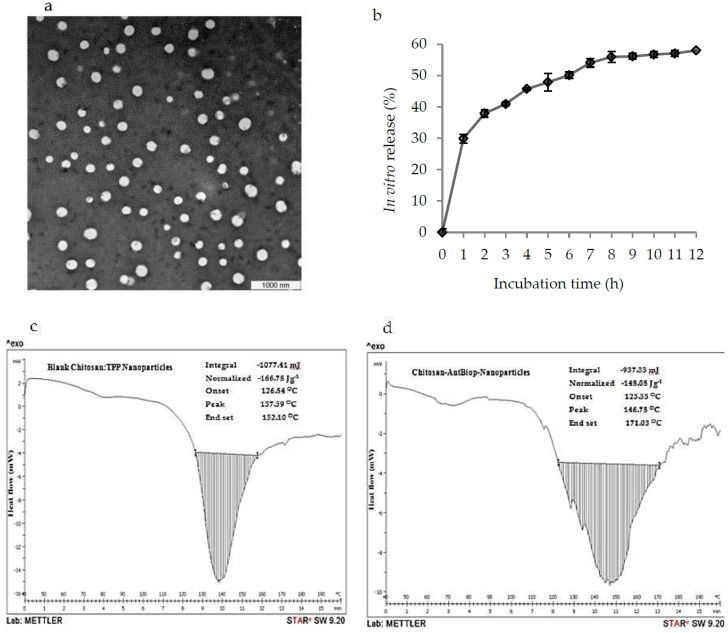
Physical appearance, in vitro release profiles and differential scanning calorimetry (DSC) of the Chit-AntBiop-NPs; (**a**) transmission electron microscopy (TEM) images of Chit-AntBiop-NPs, (**b**) In vitro release profiles of AntBiop from Chit-AntBiop-NPs in PBS pH 7.4 (**c**) DSC curve of the blank chitosan nanoparticles and (**d**) DSC curve of the Chit-AntBiop-NPs.

As shown in Figure 3a, the transmission electron microscopy (TEM) images showed that the Chitosan-AntBiop-NPs were generally homogeneous and spherical, which was supported by their narrow particle size distribution characterized by a low pdi value below 0.5.

#### 2.2.2. In Vitro Release Profiles of Chit-AntBiop-NPs

The in vitro release profile of Chitosan-AntBiop-NPs is shown in Figure 3b. The release of AntBiop encapsulated in Chit-AntBiop-NPs followed an initial phase of burst release followed by a slow phase of sustained release within the 12 h of incubation in phosphate buffered saline (PBS) pH 7.4 with a cumulative release of 58%.

The in vitro release showed a prolonged and sustained release of the AntBiop from the Chit-AntBiop-NPs into the suspending medium. This indicated the potential oral application of the Chit-AntBiop-NPs for preventing gastrointestinal degradation and improving sustained release and prolonging the therapeutic effect of the AntBiop as an antihypertensive agent.

#### 2.2.3. Differential Scanning Calorimetry (DSC)

The thermal behavior of the blank chitosan and Chitosan-AntBiop-NPs are presented in Table 1 and Figure 3c,d. A thermogravimetric analysis of the nano formulations essentially revealed their crystalline nature, which in turn affects the entrapment efficiency and release profile of the encapsulated bioactive agent. The exothermic peak in the thermograms of the chitosan-AntBiop-NPs showed a higher melting temperature of 146.78 °C compared to the control capsule at 137.59 °C, suggesting that a large amount of energy will be needed to overcome the forces of interaction between the highly organized biopeptides and their surrounding surfaces of chitosan matrix.

Similarly, the high melting temperature was reported to be due to the interaction between the active agent and its coating polymer, such that more energy is needed to dissipate their cohesive forces than that required to break the blank capsule [34].

As shown in Figure 3d, Chitosan-AntBiop-NPs have broader peaks compared to blank chitosan nanoparticles, which showed narrower peaks with a lower value of temperature difference (Table 1). The observed change in the pattern of peaks might be related to the inclusion of the AntBiop in the chitosan nanocapsules.

#### 2.2.4. In Vivo Antihypertensive Efficacy

**Figure 4:**
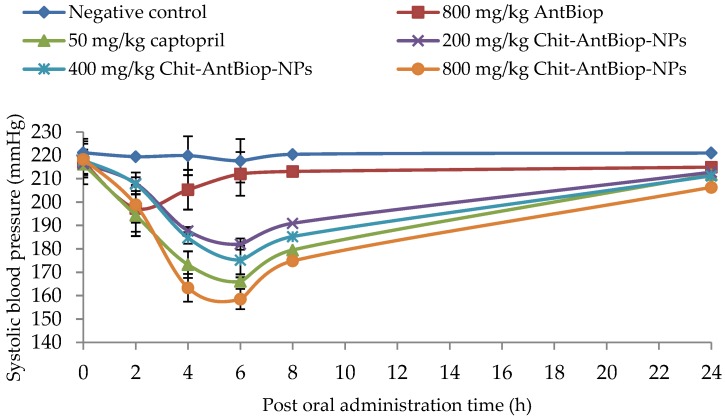
Systolic blood-pressure-lowering effect of unencapsulated biopeptides, captopril and three doses of Chitosan-AntBiop-NPs within 24 h of single oral administration on SHRs.

As shown in Table 2 and Figure 4, a single oral dose of the unencapsulated biopeptides/AntBiop, captopril and varying doses of Chit-AntBiop-NPs exhibited a systolic blood-pressure-lowering effect within 24 h of their administration. The oral administration of unencapsulated biopeptides at 800 mg/kg significantly (*p* < 0.05) reduced systolic blood pressure (SBP) by 19.78 ± 9.966 mmHg at 2 h and 11.78 ± 8.483 mmHg at 4 h respectively (Group II).

A sustained significant decrease (*p* < 0.05) in SBP between 2 and 8 h post oral administration was observed for 50 mg/kg captopril and for the 200, 400 and 800 mg/kg Chit-AntBiop-NPs. The maximum SBP-lowering effects of captopril and the Chit-AntBiop-NPs were 50.18 ± 8.90 mmHg and 59.78 ± 7.68 mmHg at 6 h post administration. Thereafter, the SBP increased gradually towards the untreated levels. A sustained decrease in SBP was still observed for the given doses of unencapsulated biopeptides, captopril and different doses of Chit-AntBiop-NPs even at 24 h of post administration. However, it was not significant (*p* > 0.05) for the unencapsulated biopeptides.

The encapsulation of the AntBiop using chitosan was found to improve their antihypertensive efficacy in a dose-dependent mode by several folds compared to un-encapsulated AntBiop (Table 2). The small size and high EE of the prepared Chit-AntBiop-NPs could have enhanced the stability, intestinal absorption and target site delivery, which might have resulted in the sustained release and long term antihypertensive effect of the encapsulated AntBiop on the SHRs. Similarly, the sustained release and enhanced in vivo efficacy of peptides and protein have been attributed to the high intestinal permeation and small size of their nanocarriers [17,35,36].

## 3. Materials and Methods

### 3.1. Materials

Low molecular weight chitosan and sodium tripolyphosphate (purity 85%) were purchased from Sigma-Aldrich, Co. (St. Louis, MO, USA). Tween 80 was purchased from Merk Schuchardt OHG (Hohenbrunn, Germany). All other reagents and chemicals used were of analytical grade and were obtained from Merk KGaA (Darmstadt, Germany) and Sigma-Aldrich, Co. (St. Louis, MO, USA).

Then, 10–15 week old male spontaneously hypertensive rats (SHRs) weighing between 250 to 320 g were obtained from the Animal Experimental Unit (AEU) Services at the Faculty of Medicine, University of Malaya, Kuala Lumpur, Malaysia. The proposed animal utilization protocol was approved by the institutional Animal Care and Use Committee, Universiti Putra Malaysia (AUP No: R078/2015, 21 January 2015).

### 3.2. Preparation of ACE-Inhibitory/Anti-Hypertensive Biopeptides (AntBiop) from Stone Fish Using Bromelain

The AntBiop were prepared as previously described by Auwal et al. [37,38]. Briefly, the lyophilized sample of stone fish was ground in a warring blender and 10 g of the powder was dialyzed in a 12–14 kDa molecular weight cut-off dialysis tube for 4 h against deionized water and 20 h against phosphate buffer pH 7 prepared at 50 mM. The dialyzed sample was then hydrolyzed with bromelain according to its previously established optimum condition defined by pH 7, temp 40 °C, Enzyme/Substrate Ratio 2% and a hydrolysis time of 240 min. The reaction mixture was boiled for 10 min at 100 °C to inactivate the enzyme and centrifuged for 10 min at 10,000× *g*. The supernatant was then lyophilized to obtain the ACE-inhibitory biopeptides with molecular weight (mw < 10 kDa) and stored at −40 °C before analysis.

### 3.3. Preparation of the Chitosan-Antihypertensive Biopeptides Nanoparticles (Chit-AntBiop-NPs)

The Chit-AntBiop-NPs were produced by the ionotropic gelation method under the optimum condition of AntBiop:chitosan mass ratio, stirring speed (rpm) and stirring time (min). Briefly, the AntBiop and chitosan were both prepared at a concentration of 0.5% *w*/*v*. The surfactant, tween 80 was also prepared at 0.5% *v*/*v* and added to the solution of the AntBiop at a volume ratio of 4:1. The AntBiop-tween 80 mixture was then transferred to the stirring solution of chitosan at a chitosan:AntBiop mass ratio of 4:1 and sheared in an IKA^®^ T 25 digital ULTRA-TURRAX^®^ high shear homogenizer (IKA^®^ Works, Inc. Wilmington, DE, USA) for 15 min. The cross-linking agent, sodium tripolyphosphate, prepared at 0.5% *w*/*v*, was then added drop-by-drop to the above solution under continuous stirring for another 15 min. The unencapsulated biopeptides (mw ˂ 10 kDa) were removed by centrifugation at 10,000× *g* for 10 min. The resulting Chit-AntBiop-NPs were ice-bath sonicated for 10 min and filtered through a 0.45 µm membrane filter before storage at 4 °C. The blank chitosan nanoparticles were similarly prepared to serve as a control.

### 3.4. Characterization of the Chit-AntBiop-NPs

#### 3.4.1. Particle Size, Polydispersity Index (pdi) and Zeta Potential (*ζ*)

The average particle size, pdi and *ζ* were measured at 25 °C in a Zetasizer (Zetasizer Nano-ZS 90; Malvern Instruments Ltd., Malvern, UK). The Chit-AntBiop-NPs suspension was diluted in deionized water and the measurements were recorded as mean ± standard deviation of five experiments.

#### 3.4.2. Entrapment Efficiency of the Nanocapsules

The amount of the biopeptides entrapped within the matrix of the chitosan nanocapsules was determined as follows. The unencapsulated AntBiop in the supernatant was quantified using a Bichinchoninic acid micro protein assay kit obtained from Sigma-Aldrich, Spruce St., St. Louis, MO, USA. The amount of the entrapped AntBiop was then determined from the total amount initially added according to the following equation:

$$\%\;EE=\frac{Total\;amount\;of\;AntBiop-Untrapped\;AntBiop}{Total\;amount\;of\;AntBiop}\times 100$$

#### 3.4.3. Morphology

The physical appearance of the Chit-AntBiop-NPs was studied by transmission electron microscopy (TEM, H-600, Hitachi, Tokyo, Japan). The samples were prepared by dilution with deionized water. After negative staining with 1% uranyl acetate solution for 1 min 30 s, a thin film of the sample was then mounted on to a grid of copper electron microscopy for visualization.

#### 3.4.4. Differential Scanning Calorimetry (DSC)

Both the control and Chit-AntBiop-NPs were freeze dried and their thermal behavior was examined by differential scanning calorimetry using a digital scan colorimeter DSC instrument (Mettler Toledo DSC821e, Schwerzenbach, Switzerland). Each sample was weighed into an aluminum pan and hermetically sealed. An empty pan was used as a reference and the thermograms were recorded within a temperature range of 40–200 °C at the heating rate of 10 °C/min.

The temperature difference *T*_f_ − *T*_o_, was calculated from the values of the end temperature (*T*_f_) and onset temperature (*T*_o_).

#### 3.4.5. In Vitro Release

The in vitro release profile of AntBiop from Chit-AntBiop-NPs was monitored by dialysis in a 12–14 kDa molecular weight cut-off (MWCO) dialysis tube against phosphate buffered saline pH 7.4. The Chit-AntBiop-NPs and the control capsule were both transferred in to the dialysis tube and suspended in the release media at 37 °C with shaking at 250 rpm for 12 h. Then, 1 mL of the Chit-AntBiop-NPs was withdrawn at 1 h interval and the amount of the released AntBiop was measured as in Section 3.4.2 and expressed as the change in entrapment efficiency with time according to the following formula:

In vitro release (%) = [(*E*_0_ − *E_i_*)/*E_i_*] × 100

where *E*_0_ stands for the entrapment efficiency at a time *t* = 0 before the start of dialysis and *E_i_* is the entrapment efficiency at a time *t* = *i* (where *i* is 1 to 12 h) during the course of the dialysis.

#### 3.4.6. In Vivo Antihypertensive Effect

The in vivo antihypertensive efficacy of the Chit-AntBiop-NPs was evaluated on SHRs. The study involved a total of 36 rats which were assigned into six groups of six rats each. They were acclimatized for one week in a controlled temperature room (22 °C) with a 12 h light/dark cycle and allowed free access to water and a standard commercial laboratory diet (Altromin Spezialfutter GmbH & Co. KG–Im Seelenkamp 20, 32791 Lage, Germany). The rats in each group were orally administered once with single dose by oral gavage. Group I (negative control) received blank Chit-NPs (800 mg/kg, 3.2 mL/kg), Group II (positive control) were given captopril (50 mg/kg, 2.5 mL/kg), group III were administered with unencapsulated biopeptides/AntBiop (800 mg/kg, 3.2 mL/kg) while groups; IV, V and VI were treated with varying doses of Chit-AntBiop-NPs (200 mg/kg, 0.8 mL/kg; 400 mg/kg, 1.6 mL/kg, and 800 mg/kg, 3.2 mL/kg). The blank chitosan nanoparticles and Chit-AntBiop-NPs were dissolved in normal saline and 0.1 N HCl before administration. The volume of each dose given was determined based on body weight of the SHRs.

The rats were placed in a holder, then pre-warmed to 35 °C on a heating flat form and the blood pressure was measured at 0 h before administration and then at 2, 4, 6, 8 and 24 h after administration to evaluate the SBP-lowering effect due to each dose using a non-invasive tail cuff device (Kent Scientific Corporation, Toorington, CT, USA).

#### 3.4.7. Statistical Analysis

The statistical analysis was carried out using Minitab 16.0 software (MINITAB, State College, PA, USA). Data analysis was carried out using one-way analysis of variance (ANOVA). A difference at *p* <0.05 was taken to be statistically significant.

## 4. Conclusions

The Chit-AntBiop-NPs were fabricated under the optimum condition of AntBiop:Chitosan mass ratio, stirring speed and stirring time. The optimized capsules showed homogenously dispersed and small-sized particles of 162.47 nm with a high entrapment efficiency of 75.36%. The biopeptide- containing capsules showed a higher melting temperature of 146.78 °C compared to 137.59 °C for the blank capsules, with a cumulative release of 58% after 12 h in PBS pH 7.4. Furthermore, the in vivo antihypertensive efficacy indicated a significant (*p* < 0.05), dose-dependent and sustained blood-pressure-lowering effect of the Chitosan-AntBiop-NPs, which was prolonged up to 24 h. Thus, the chitosan coating improved the gastrointestinal stability and bioavailability of the AntBiop. This demonstrated the relevance of the AntBiop-Chitosan-NPs for the management of hypertension.

## Figures and Tables

**Table 1:** Changes in the enthalpies obtained from DSC thermograms of blank Chitosan nanocapsules and Chit-AntBiop-nanocapsules.

S/N	Formulation	Peak Type	Temperature (°C)				Normalized Δ*H* (J/g)	Integral (mJ)
			Onset (*T*_o_)	Peak (*T*_p_)	End Set (*T*_f_)	Δ*T* (*T*_f_ − *T*_o_)		
**1**	Blank chitosan nanocapsules	Exothermic	126.84	137.59	152.10	25.26	−166.78	−1077.41
**2**	Chit-AntBiop-nanocapsules	Exothermic	123.55	146.78	171.03	47.48	−148.00	−937.33

DSC: digital scan colorimetry; AntBiop: antihypertensive biopeptides; *T*_o_: Onset temperature; *T*_p_: Peak temperature; *T*_f_: End or final temperature; Δ*T*: Temperature change/difference.

**Table 2:** The magnitude of systolic blood pressure reduction due to a single oral dose on spontaneously hypertensive rats (SHRs) as mean ± standard deviation (mmHg) within 24 h post administration.

Time (h)	Negative Control (Group I) *n* = 6	800 mg/kg AntBiop (Group II) *n* = 6	50 mg/kg Captopril (Group III) *n* = 6	200 mg/kg Chit-AntBiop-NPs (Group IV) *n* = 6	400 mg/kg Chit-AntBiop-NPs (Group V) *n* = 6	800 mg/kg Chit-AntBiop-NPs (Group VI) *n* = 6
**2**	1.68 ± 4.168	19.78 ± 9.966 *	21.92 ± 6.668 *	8.44 ± 0.775 *	10.22 ± 3.415 *	19.39 ± 4.474 *
**4**	1.25 ± 0.970	11.78 ± 8.483 *	43.06 ± 8.642 *	28.77 ± 4.377 *	33.22 ± 3.102 *	54.95 ± 7.703 *
**6**	3.44 ± 0.515	5.01 ± 8.033	50.18 ± 8.901 *	34.46 ± 4.618 *	42.68 ± 2.931 *	59.78 ± 7.682 *
**8**	0.68 ± 1.322	3.95 ± 5.461	36.77 ± 5.708 *	25.55 ± 1.627 *	32.62 ± 2.468 *	43.45 ± 5.956 *
**24**	0.13 ± 0.104	2.12 ± 9.323	4.5 ± 1.721 *	3.69 ± 2.383 *	6.66 ± 6.116 *	11.96 ± 4.349 *

Statistical significance was calculated using one-way analysis of variance (ANOVA) at a level of *p* < 0.05 and is denoted by asteric superscript (*) for both AntBiop group, captopril group and three different Chit-AntBiop-Nps groups versus the negative control group.
